# Clinical Identification of Two Novel *C. kroppenstedtii*-like Species Isolated as Pathogens of Granulomatous Lobular Mastitis

**DOI:** 10.3390/pathogens13100880

**Published:** 2024-10-09

**Authors:** Nan Xiao, Xiu-Ying Zhao

**Affiliations:** Department of Laboratory Medicine, Beijing Tsinghua Changgung Hospital, School of Clinical Medicine, Tsinghua University, Beijing 100190, China

**Keywords:** *C. kroppenstedtii*, granulomatous lobular mastitis (GLM), *rpoB*, Mass Spectral Peaks (MSPs), antibiotic sensitivity tests (AST)

## Abstract

Granulomatous lobular mastitis (GLM) is a rare benign breast inflammatory disease that affects women of childbearing age. *Corynebacterium* species, especially *Corynebacterium kroppenstedtii*, was reported as the pathogen of GLM. A recent study showed that the *C. kroppenstedtii* complex is composed of *C. kroppenstedtii* and two novel species, *C. parakroppenstedtii* and *C. pseudokroppenstedtii*. The study presents seven *C. kroppenstedtii*-like strains isolated from GLM patients. However, they turned out to be six strains of *C. parakroppenstedtii* and one strain of *C. pseudokroppenstedtii* according to 16sRNA sequencing. In order to conduct a phylogenetic study, we further sequenced the fusA and *rpoB* genes, which were frequently employed in studies of *Corynebacterium* species. Novel Mass Spectral Peaks (MSPs) for *C. parakroppenstedtii* were created with Bruker MALDI-TOF MS. Then, the identification power of the MSPs was tested by *C. parakroppenstedtii* strains and remotely related *Corynebacterum* spp. The antibiotic sensitivity tests were performed according to the CLSI M45 guidelines. All of the strains were not resistant to *β*-lactams, vancomycin or linezolid. However, applying erythromycin and clindamycin could be fruitless. Phenotypic identification using a Vitek2 ANC ID card proved all of the *C. parakroppenstedtii* strains were identified as *Actinomycete naeslundii*. The test of Ala-Phe-Pro arylamidase and urease could be employed as the characteristics to distinguish *C. pseudokroppenstedtii* from *C. parakroppenstedtii*. Here, we present the identification, antibiotic sensitivity tests (ASTs) and epidemiological investigation of two novel *C. kroppenstedtii*-like species with the purpose of improving the understanding of *C. kroppenstedtii*-like species and related diseases.

## 1. Introduction

Granulomatous lobular mastitis (GLM) is a rare benign breast inflammatory disease that characteristically affects young and middle-aged women, especially who have a recent history of pregnancy and lactation [1]. In 2003, a review of 34 patients in New Zealand reported a strong association between GLM and *Corynebacterium* species [2]. From then on, the reports of the bacterial pathogen kept emerging. In Mediterranean countries and in China, the incidence of GLM has increased rapidly. In China, it was estimated that at least 10,000 individuals were annually affected by GLM [3]. For some patients, it is even worse since the treatment may be complicated and prolonged symptoms may take place, which seriously affects the quality of life of young women.

Researchers believed that several factors might play a role in the pathogenesis of GLM, such as immune dysregulation, hormonal components and bacterial infection [3,4]. Recently, people became interested by the complicated relationship between GLM and bacterial pathogens. Some recent studies indicated that GLM was associated with lipophilic *Corynebacterium* species, such as *Corynebacterium kroppenstedtii* and to a less extent *Corynebacterium tulerculostearicum* [5,6].

The genus *Corynebacterium*, which consists of more than 130 species, represents a group of Gram-positive, non-spore-forming, rod-shaped bacteria phylogenetically assigned to the order *Corynebacteriale* and family *Corynebacteriaceae* [7]. Previous studies tend to assign *Corynebacterium* spp. as opportunistic pathogens. These Gram-positive rod-shaped bacillus are usually dismissed as contaminants or normal flora when isolated from clinical specimens since they are common components of the skin microbiome. Nevertheless, they have been increasingly recognized as being associated with a variety of diseases [8]. *C. kroppenstedtii* was first isolated in 1998 from a human sputum specimen [9]. Now, people have recognized that *C. kroppenstedtii*-like bacterium is associated with breast abscesses and granulomatous mastitis [10]. The growth rate of *C. kroppenstedtii* in the conventional medium employed in most clinical laboratories was usually unsatisfactory [11]. Recently, newly developed diagnostic techniques, including matrix-assisted laser desorption ionization-time of flight mass spectrometry (MALDI-TOF MS) and 16S rRNA sequencing, have been adopted by clinical laboratories globally, and more and more GLM cases associated with *C. kroppenstedtii* infection have been reported [12,13].

Contemporary studies showed that the *C. kroppenstedtii* complex is composed of *C. kroppenstedtii* and two novel species, *C. parakroppenstedtii* and *C. pseudokroppenstedtii* [14]. In this study, seven non-repetitive *C. kroppenstedtii* complex strains were identified from routine bacterial cultures with pus samples from GLM patients. The biological characteristic of these isolates and the clinical features of the cases were analyzed. The study of the ability of identifying the *C. kroppenstedtii* complex with MALDI-TOF-MS and Vitek ID Card system was also conducted.

## 2. Materials and Methods

### 2.1. Study Design and the Enrollment of the Patients

This study enrolled GLM patients presenting to the outpatient department of Beijing Tsinghua Changgung Hospital, a tertiary hospital in the northern part of Beijing, from January 2022 to December 2023. All the patients enrolled in this study had been diagnosed as GLM by Doppler ultrasound with or without pathologic tests. All of them underwent incision or puncture drainage of the abscess. After the operation, the samples of pus were sent to our laboratory for the bacterial culture. If the culture showed no bacteria growth, then the patients would be excluded.

The samples were cultured on Columbia blood agar plates for up to 5 days of incubation with 35 °C and 5% CO_2_ atmosphere. If there were few colonies that grew slowly and were grayish, smooth, circular, and nonhemolytic, the colonies would undergo identification by Bruker MALDI-TOF-MS. If the colonies were identified as *Corynbacterium* spp., the patient would be enrolled. All the *Corynbacterium* spp. strains would be numbered in time sequence with the numbering system of the strain bank in the strain bank of the laboratory.

The demographic data of the patients included age, the white blood cell (WBC) count, the value of the C-reaction protein (CRP), the size of the main breast abscess, whether or not there were multiple abscesses, the possible enlargement of axillary lymph nodes, and related medical history.

### 2.2. Identification of C. kroppenstedtii-like Strains from 16S rRNA Sequencing

The 16S rRNA genes of all seven strains were PCR-amplified using the universal primers 27f and 1492r [15] and the ready-to-use kit One Taq Quick Load 2xMM with Standard Buffer (Takara Bio Inc, Beijing, China). The PCR reaction mix consisted of 10 μL of the ready-to-use kit, 37.5 pmol of each primer, 1 μL of DNA and 9 μL of PCR grade water. The amplification program was set at an initial denaturation step at 95 °C for 5 min, which was followed by 30 cycles of denaturation at 94 °C for 1.5 min, annealing at 55 °C for 1.5 min and elongation at 72 °C for 5 min, and it was finished by a final elongation at 72 °C for 10 min. The resulting PCR products were purified for Sanger sequencing technology. The 16S rRNA sequences were analyzed by the online platform EZbiocloud (http://www.ezbiocloud.net/ (accessed on 9 March 2024))

### 2.3. Analysis of the Phylogenetic Relationships

For more detailed genetic analysis, partial gene sequences of translation elongation factor EF-G (*fusA*) [16] and partial sequences of the RNA polymerase beta subunit-encoding gene *rpoB* [17] of the 7 isolates were subjected to PCR amplification. The hypervariable region of the *rpoB* gene was sequenced with the reported primers C2700F and C3130R [17]. The PCR mixtures were subjected to 35 cycles of denaturation at 94 °C for 30 s, with primer annealing at 58 °C for 30 s, and then it was extended at 72 °C for 2 min. The PCR amplification reaction was ended with a final elongation step of 72 °C for 10 min. The resulting PCR products were purified for Sanger sequencing technology just like the 16S rRNA sequences. The primers employed in the PCR reaction are listed in Table 1. The sequences were analyzed and OrthoANI values were calculated by the online platform EZbiocloud (http://www.ezbiocloud.net/). The phylogenetic trees of the *C. kroppenstedtii*-like strains were drawn with maximum-likelihood model.

### 2.4. Creating Novel Database Entries Known as Mass Spectral Peaks (MSPs) with Bruker MALDI-TOF-MS System Specific for C. parakroppenstedtii

We created novel MSPs for *C. parakroppenstedtii* since this newly found strain was not included in the database known as Bruker Taxonomy Projects provided by the producer. The instructions from the producer were followed. In brief, a calibration of the machine should be made first. For the database entry, the strains have to be prepared with the formic acid extraction method to ensure a high quality of spectra. With the 6 isolates of the *C. parakroppenstedtii*, three spots for each isolate were prepared on the target schema. Baseline subtraction and the smoothing of each spectrum were performed with the software flexAnalysis 3.4. Then, we added the controlled spectra into the software Biotyper 3.1 to create a novel MSPs for *C. parakroppenstedtii*. Then, the newly recovered clinical strains of *C. parakroppenstedtii*, *C. pseudokroppenstedtii*, *C. glucuronolyticum* and *C. jeikeium* from our laboratory were employed to verify the identification power of the novel MSPs.

### 2.5. Antibiotic Sensitivity Tests (AST)

The broth microdilution method (BMD) was performed according to the CLSI M45 3rd Edition guidelines for aerobic *Actinomycetes* and *Corynebacterium* species. Briefly, with a sterile swab, colonies were swabbed from a blood agar plate (48 to 72 h) and transferred to 3 mL sterile normal saline. The inoculum was then vortexed for more than 20 s in order to eliminate any remaining clumps. The concentration of the suspension achieving a 0.5 McFarland standard was required. For the broth microdilution method, 0.5 mL of the 0.5 McFarland suspension was added to 4.5 mL sterile saline (1:10 dilution), resulting in ∼10^7^ CFU/mL. Microtiter plates (96 well) were commercial kits (Bio-kont Ltd., Wenzhou, China). The antimicrobial agents tested included penicillin (0.064–8 μg/mL), clindamycin (0.016–32 μg/mL), ceftriaxone (0.5–64 μg/mL), ciprofloxacin (0.064–8 μg/mL), gentamicin (0.25–32 μg/mL), erythromycin (0.125–16 μg/mL), meropenem (0.125–16 μg/mL), vancomycin (0.5–64 μg/mL), and linezolid (0.25–32 μg/mL). Microtiter plates were sealed in a homemade wet box and incubated at 35 °C. The MICs were read at 24, 48, and 72 h. If required due to poor growth, plates were re-incubated for a further 48 h, and a final MIC reading was made on day 5 (120 h).

### 2.6. Phenotypic Testing

Previous studies have confirmed that the Vitek 2 ANC ID card (bioMérieux, France) is a simple, rapid, and satisfactory method applied in clinical microbiology laboratories to identify anaerobes and Gram-positive bacteria [18]. In China, the Vitek 2 system is one of the most widely used automated microbiology systems in tertiary hospitals all over the country. We employed the Vitek 2 system to perform the phenotypic tests. Bacterial colonies were suspended in 0.45% sodium chloride with a turbidity of 3.5–4.0 McFarland. Inoculums were then introduced into an ANC ID card in the Vitek 2 Compact automated identification system and incubated for approximately 6 h. The system would analyze the bacterial growth and whether or not the bacterium reacts with a serial of biochemical reagents in the little transparent cells of the ID card. The matrix of the reaction rates leads to the identification of different bacteria. The results of the biochemical reactions tested reflected the phenotypic characteristics of the bacterium.

### 2.7. Ethics Statement

This study complies with all applicable international and domestic ethical regulations. Ethical approval by Ethics Committee of Beijing Tsinghua Changgung Hospital was issued with the number of 23495-0-01. All participants signed informed consent forms and were informed that they could withdraw from the study at any time. The collection and processing of research data complied with privacy protection principles, and all identifiable personal information has been deleted or encrypted.

## 3. Results

### 3.1. The Demographic and Medical Data of the GLM Patients with C. kroppenstedtii-like Bacterium Infection

Seven strains of *C. kroppenstedtii-like* Gram-positive rod-shaped bacteria were isolated from GLM patients. They were all identified as *C. kroppenstedtii* by Bruker MALDI-TOF-MS with scores from 1.50 to 1.95. The *C. kroppenstedtii*-like strains were numbered as 7403, 7413, 7991, 8117, 8226, 8285, and 8318, as were the patients. All the demographic and medical data of the GLM patients are listed in the Table 2. The ages of the patients were from 30 to 39 years old. Only patient no. 8226 suffered from the dilation of mammary ducts, while the others all had abscesses. All the patients were diagnosed with GLM by Doppler ultrasonic examination, while only no. 7991 and no. 8117 were subject to pathology tests. Three of seven patients suffered multiple abscesses. Except for one patient with no records of blood test, five of six patients had WBC counts higher than 10 × 10^9^/L, and when the diameters of the main abscess were greater than or equal to 7.0 cm, the patients would have enlarged axillary lymph nodes. Three patients had possible risk factors for GLM. Patient no. 7403 had a history of lactation. Patient no. 7991 reported trauma of the breast 2 months before being diagnosed as GLM. Patient no. 8226 reported being pregnant for 9 weeks. Four reported no obvious cause. All of them underwent conservative treatments such as applying external antibiotic ointments but failed. After the incision or puncture drainage of the abscess, most of them healed successfully with the help from cephalosporin antibiotics. Only patient no. 7991 suffered a long period of recurrent infections of the breast tissue. She went to the surgical outpatient department for several times because of the GLM. The ordinal treatment seemed invalid.

### 3.2. Identification of the C. kroppenstedtii-like Strains and Phylogenetic Analysis

**Figure 1:**
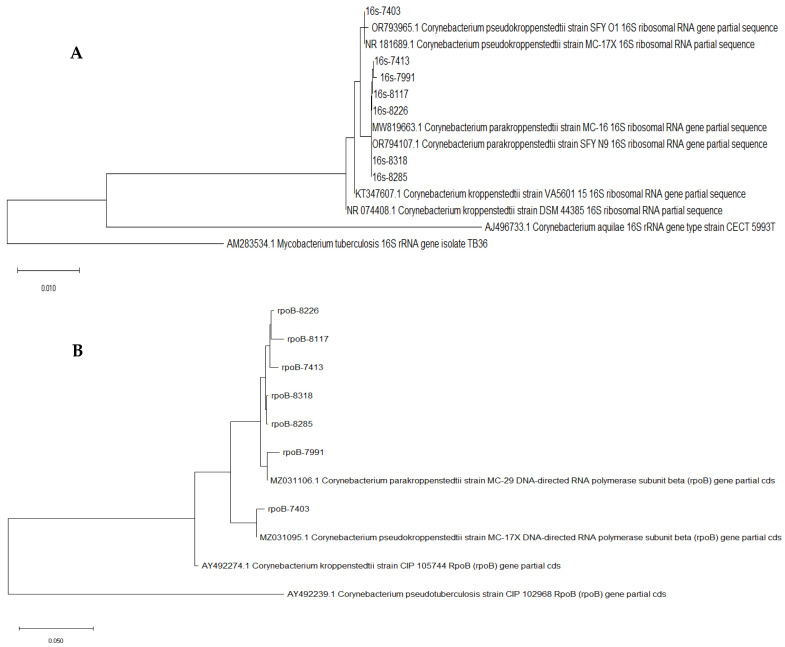
Maximum-likelihood trees for the 7 *C. kroppenstedtii*-like strains. (**A**) Phylogenic tree A by 16sRNA; (**B**) phylogenetic tree B by the partial *rpoB* gene.

All the 16sRNA sequences were analyzed with the online platform EZbiocloud [19]. Six of the seven *C. kroppenstedtii*-like isolates were *C. parakroppenstedtii* while only no. 7403 was identified as *C. pseudokroppenstedtii*. None of the isolates was *C. kroppenstedtii*. The OrthoANI values among the 16S rRNA sequences and partial fusA gene sequences were from 99.33% to 100.0% and from 97.21% to 100%, respectively, by the ANI calculator of the platform EZbioCloud [19]. The OrthoANI values among the partial *rpoB* gene were from 94.38% to 99.57%. The discriminating ability of the partial *rpoB* gene is better than that of the 16S rRNA sequences and the partial *fusA* gene in the C. kroppenstedtii-like species. The maximum-likelihood trees were drawn with the software MEGA 11.0 [20]. Phylogenic tree A was drawn by 16sRNA sequences (Figure 1A), while phylogenetic tree B was drawn by the partial rpoB gene (Figure 1B). The other sequences of 16S rRNA and *rpoB* of the strains of *Corynebacterum* spp. used as references in the trees were downloaded from the NCBI Genebank database.

### 3.3. Novel MSPs for C. parakroppenstedtii

**Figure 2:**
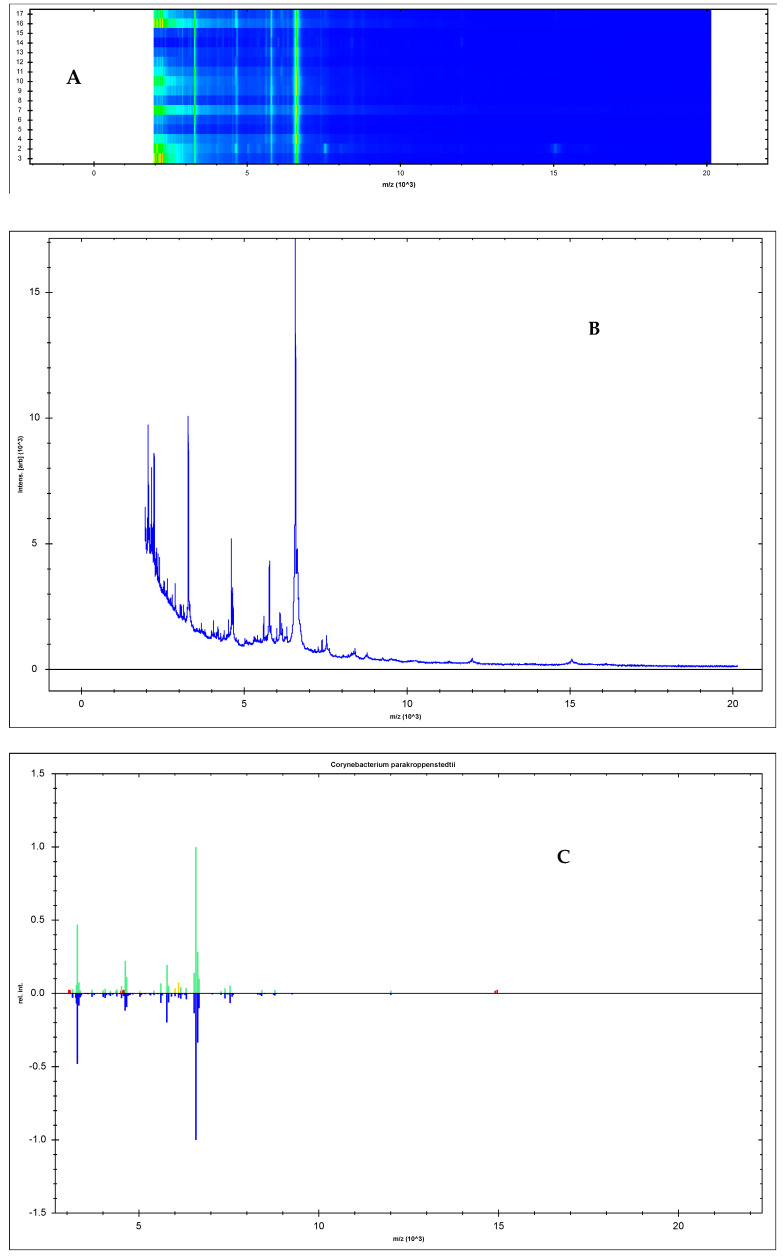

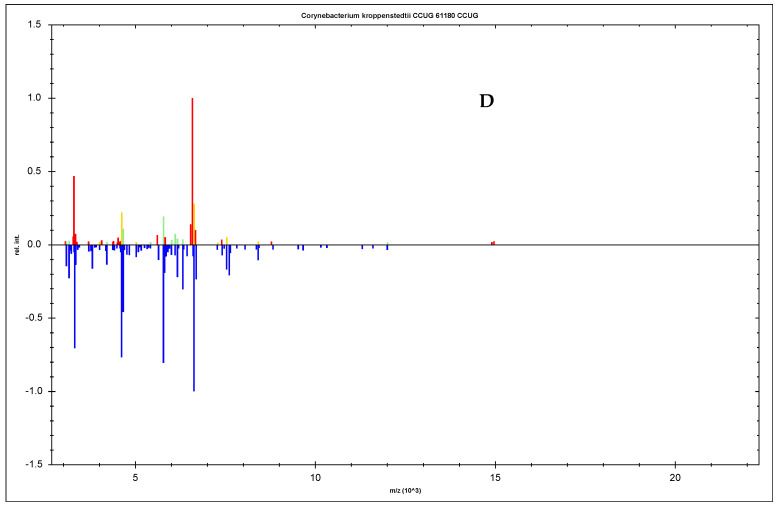
(**A**) Gelview of the spectra of *C. parakroppenstedtii* isolates; (**B**) spectrum of *C. parakroppenstedtii*; (**C**) novel MSPs for *C. parakroppenstedtii*; (**D**) novel MSPs compared with that of *C. kroppenstedtii.* Red color of the peaks means the unmatched one while the yellow color means better match. Green color is the perfect match.

Since there were six different *C. parakroppenstedtii* isolates, the number was enough to create brand new MSPs for this species, which were not included in the Bruker Taxonomy Projects database. The gelview, the spectra and the MSPs are indicated in Figure 2A–C. Figure 2D shows the differences between the MSPs of *C. parakroppenstedtii* and *C. kroppenstedtii* provided by Bruker.

**Figure 3:**
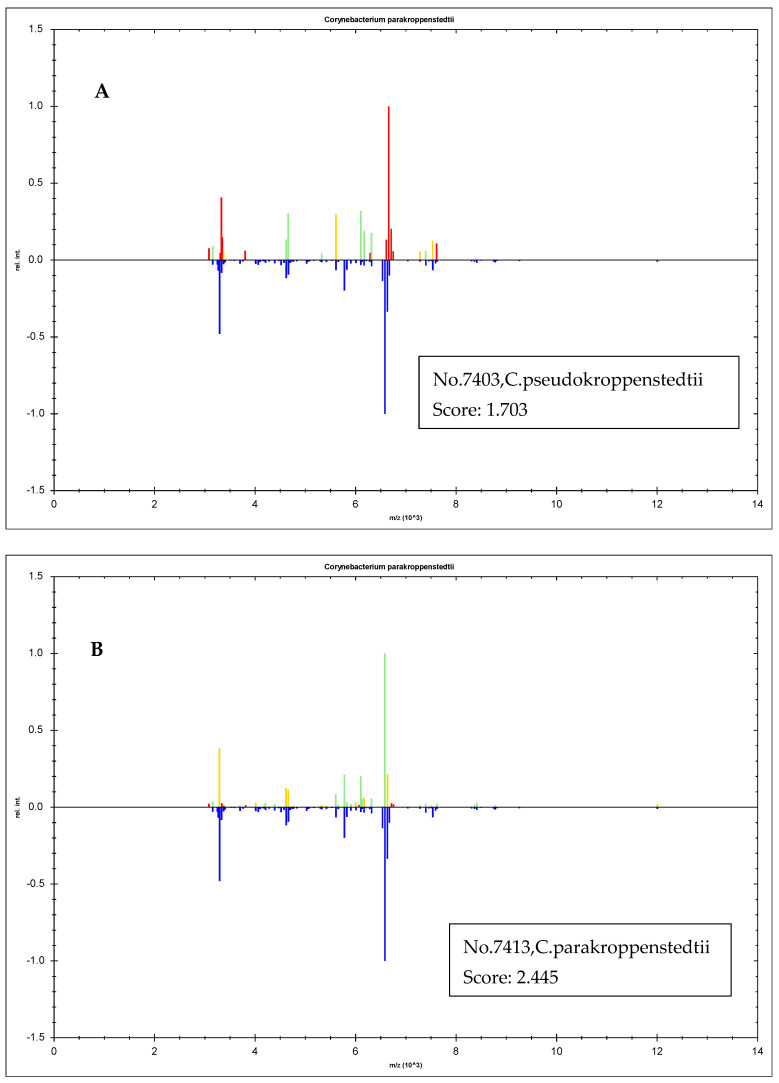

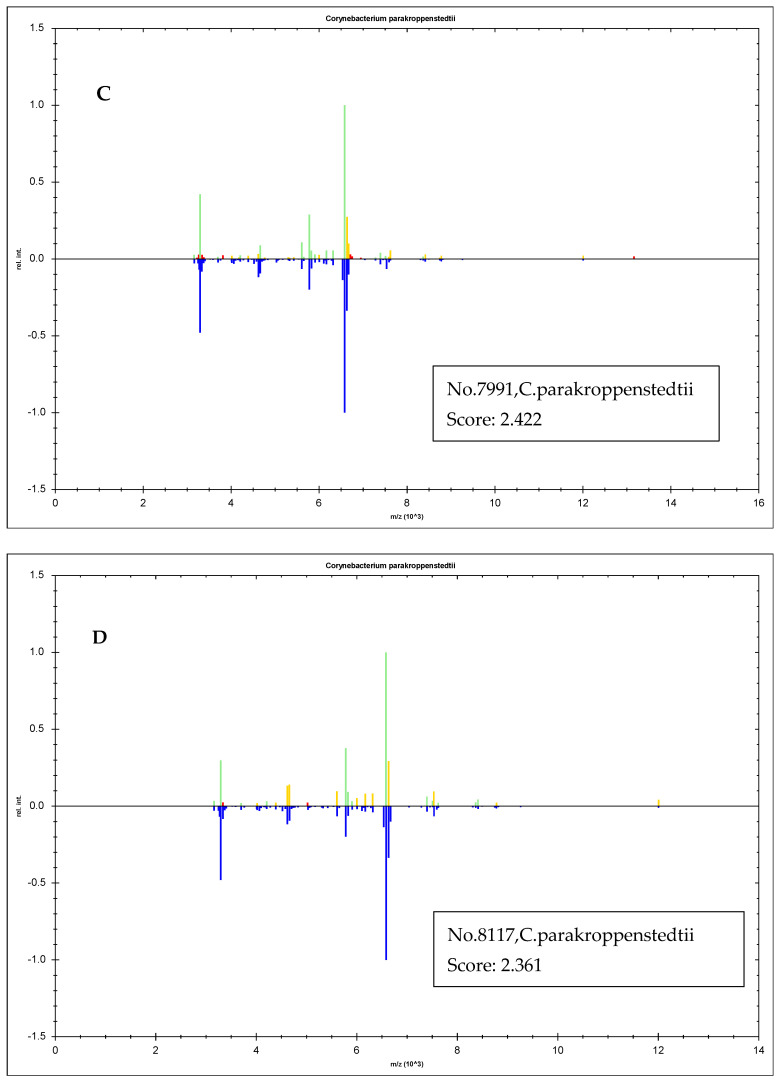

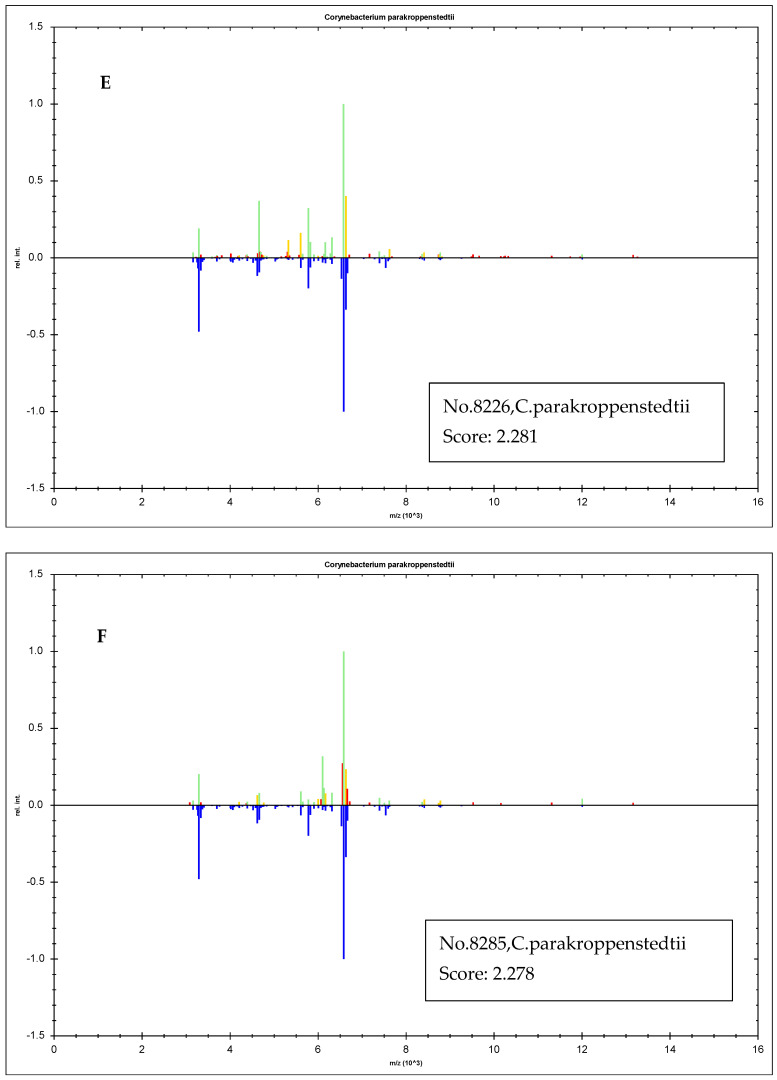

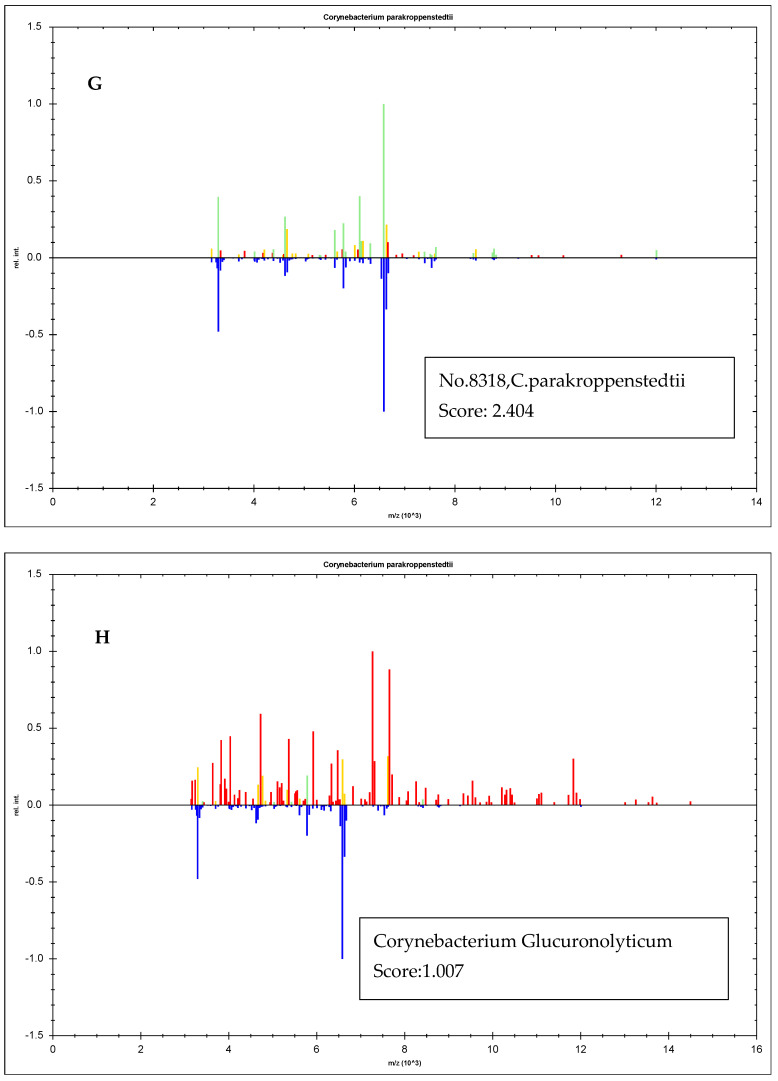

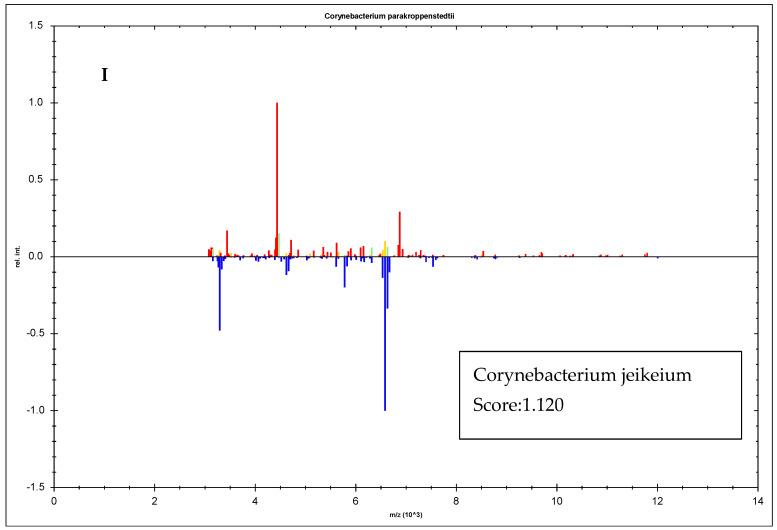
The identification power of the new MSPs established for *C. parakroppenstedtii* when it was employed in the identification of four different *Corynebacterium* spp. (**A**) The identification score of the *C. pseudokroppenstedtii* strain numbered as no. 7403 with the new MSPs was 1.703; (**B**) the identification score of the *C. parakroppenstedtii* strain numbered as no. 7413 with the new MSPs was 2.445; (**C**) the identification score of the *C. parakroppenstedtii* strain numbered as no. 7991 with the new MSPs was 2.422; (**D**) the identification score of the *C. parakroppenstedtii* strain numbered as no. 8117 with the new MSPs was 2.361; (**E**) the identification score of the *C. parakroppenstedtii* strain numbered as no. 8226 with the new MSPs was 2.281; (**F**) the identification score of the *C. parakroppenstedtii* strain numbered as no. 8285 with the new MSPs was 2.278; (**G**) the identification score of the *C. parakroppenstedtii* strain numbered as no. 8318 with the new MSPs was 2.404; (**H**) the identification score of a strain of *Corynebacterium glucuronolyticum* with the new MSPs was 1.007; (**I**) the identification score of a strain of *Corynebacterium jeikeium* with the new MSPs was 1.120. Red color of the peaks means the unmatched one while the yellow color means better match. Green color is the perfect match.

The identification power of the new MSPs created for *C. parakroppenstedtii* was validated (Figure 3A–I) when it was applied to the identification of different species of *Corynebacterium* spp. including *C. pseudokroppenstedtii*, *C. parakroppenstedtii*, *C. glucuronolyticum* and *C. jeikeium*.

The identification score of *C. parakroppenstedtii* was from 2.445 to 2.278 (Figure 3B–G), while that of the closely related species *C. pseudokroppenstedtii* was 1.703 (Figure 3A). When the species with distant genetic relationships, such as *C. glucuronolyticum* and *C. jeikeium*, were analyzed with the new MSPs, the identification scores could be as low as 1.007 and 1.210 (Figure 3H,I).

The intensities and frequencies of the most frequent *m*/*z* (Da) peaks of the new MSPs for *C. parakroppenstedtii* can found in the Supplementary Materials.

### 3.4. AST Results

Based on the CLSI breakpoints for this genus, most isolates of *C. kroppenstedtii*-like isolates were sensitive to meropenem, ceftriaxone, penicillin, vancomycin, gentamicin, and linezolid. All of them were resistant to clindamycin and erythromycin. Almost half of them (3/7) were resistant to ciprofloxacin (Table 3).

### 3.5. Phenotypic Analysis Results

All the six *C. parakroppenstedtii* strains were identified automatically as *Actinomycete naeslundii*, while the identification of the only *C. pseudokroppenstedtii* isolate failed. The reaction results are listed in Table 4. Ala-Phe-Pro arylamidase and urease could be employed as the characteristics to distinguish *C. pseudokroppenstedtii* from *C. parakroppenstedtii*. Ala-Phe-Pro arylamidase and urease tests were negative in all the six *C. parakroppenstedtii* strains while they were positive in the only *C. pseudokroppenstedtii* strain no. 7403. The tests of L-arabinosea, D-ribose and D-xylose reported a 66.7% positive rate among *C. parakroppenstedtii* strains. The tests of D-galactose, leucine arylamidase, ELLMAN, phenylalanine arylamidase, L-proline arylamidase, D-cellobiose, tyrosine arylamidase, D-glucose, D-mannose, D-maltose, saccharose/sucrose, arbutin, N-acetyl-D-glucosamine and maltotriose were 100% positive among all *C. kroppenstedtii*-like strains (Table 4).

## 4. Discussion

Since the association between GLM and some lipophilic *Corynebacterium* species was discovered for the first time in 1996 [21], there has been more and more research focused on the clinical importance of this group of rod-shaped bacteria, especially *C. kroppenstedtii* [22,23,24,25]. It was notable and understandable that the *C. kroppenstedtii* was the only *C. kroppenstedtii* complex species included in the database of Bruker MALDI-TOF MS, which was the only method we could use to identify this species, since the Vitek ANC ID card seemed unreliable in our study. Now, we found out that none of the seven “*C. kroppenstedtii*” strains were real *C. kroppenstedtii*. Most of them were actually *C. parakroppenstedtii* and only one strain was *C. pseudokroppenstedtii*. These two novel *C. kroppenstedtii*-like species were recently introduced [14]. This finding explained why the identification scores of the “*C. kroppenstedtii*” strains could never be as high as 2.00, which meant an excellent identification in the Bruker system.

Recent data suggested that hyperprolactinemia may play an important role in the development of mastitis caused by *C. kroppenstedtii* [12]. Prolactin was thought to modulate the inflammatory response and play a role in mastitis pathogenesis [26]. However, only one patient reported a history of lactation while another patient was pregnant. Pregnancy may affect the mammary glands. Most of the infection cases had no obvious causes. All the patients underwent incision or puncture drainage of the abscess, since the conservative treatment failed. After the operation, most of them healed successfully with the treatment of the cephalosporin. Only patient no.7991 who reported a history of breast trauma with the highest level of WBC count, CRP value and multiple abscesses kept attending hospital because of GLM for more than one year to date.

With the OrthoANI calculations, it seemed that the hypervariable region of the rpoB gene had the highest variability compared with the 16S rRNA and partial fusA gene. Since the OrthoANIs of these two gene sequences among the *C. kroppenstedtii*-like strains could be as high as 100%, the discrimination ability of them within these close species is doubtful, though these sequences were usually employed in the identification and phylogenetic analysis of *Corynebacterium* species [16]. The maximum-likelihood tree drawn based on the hypervariable region of the *rpoB* gene showed that no.7991 has a unique position on the tree. The relationship between no.7991 and the severe illness of the patient is still unknown and needs further investigation.

Since most of the pathogens of GLM isolated in our lab were actually *C. parakroppenstedtii*, we need to establish novel MSPs for the MALDI-TOF MS device to correctly identify this species. Now, the MSPs of *C. parakroppenstedtii* have been created. The identification ability of the novel MSPs has been proved by newly recovered *C. parakroppenstedtii* and other related *Corynebacterum* spp. strains. The discrimination ability of the novel MSPs was good enough to distinguish the closely related *C. pseudokroppenstedtii* from *C. parakroppenstedtii*. All the *C. parakroppenstedtii* strains obtained scores higher than 2.200, which meant a good identification in the Bruker MALDI-TOF MS system while no other *Corynebacterum* spp. obtained a score higher than 1.703.

According to our study and another report [14], the chance of isolating *C. pseudokroppenstedtii* strains is about one sixth of that of *C. parakroppenstedtii* in China. In case of the minor chance of the future isolates of *C. pseudokroppenstedtii*, more strains will be collected in order to create new MSPs for this species.

We chose antibiotic agents according to the CLSI M45 3rd Edition guidelines. All the *C. kroppenstedtii*-like strains were not resistant to *β*-lactams, including carbapenem, 3rd generation cephalosporin and penicillin. All the strains were sensitive to vancomycin and linezolid, since they targeted Gram-positive bacteria, and 100% resistance to clindamycin and erythromycin was observed. A similar AST result was reported by other researchers [14]. Macrolide, lincosamide, and streptogramin resistance gene erm(X) were reported to be detected in most of the *C. kroppenstedtii*-like strains isolated [14]. These findings may explain the high resistance rate of such antibiotics. Since erythromycin ointment is a commonly prescribed medication in surgical outpatient clinics in China, those findings may explain some treatment failure. According to our AST results, some resistance genes against quinolone antibiotics may also exist, and further efforts are needed to uncover the mechanism.

The misidentification of *C. parakroppenstedtii* and *C. pseudokroppenstedtii as C. kroppenstedti* may not render any negative effect on clinical treatment. However, such a minor misidentification is a relatively optimistic situation in a grassroots clinical laboratory. In most cases, they could even not obtain a valid identification, since not every laboratory in China has a mass spectrometer or DNA sequencer.

The biochemical identification is the only method in most Chinese grassroot clinical laboratories. The Vitek 2 automated microbial identification system is a widely used instrument in Chinese clinical laboratories. If any Gram-positive rod-shaped strains with grayish small colony isolated from pus samples of GLM patients were automatically identified as *Actinomycete naeslundii* by the system, the laboratory personnel should be cautious. Since Actinomycosis has little in common with GLM in term of clinical course, the wrong identification results could be misleading to the physicians or surgeons who follow up the patients, especially the ones who suffered from delayed heal. If the biochemical test results were similar to the data provided in our study, it may be speculated as *C. kroppenstedtii*-like species. It was notable that the Ala-Phe-Pro arylamidase and urease tests were negative in all six *C. parakroppenstedtii* strains, while they were positive in the only *C. pseudokroppenstedtii* strain. Since the number of strains was not big enough to draw a solid conclusion, further efforts are needed to study the biochemical identification method for *C. kroppenstedtii*-like strains.

The grassroot laboratory efforts in the field need updated knowledge with these bacteria. The culture of the *C. kroppenstedti*-complex often failed because of its slow growth rate, unremarkable colony appearance and low identification ability. Laboratory staff should pay more attention to those species in order to improve the isolation rate.

In conclusion, all the *C. kroppenstedtii*-like clinical isolates associated with GLM in our lab turned out to belong to two novel species within the genus *Corynebacterium*, *Corynebacterium parakroppenstedtii* and *Corynebacterium pseudokroppenstedtii*. The identification methods in most clinical laboratories should be modified to correctly identify the novel species. We hope that our experience in the identification, AST and epidemiological investigation could help colleagues and doctors improve their understanding of the *C. kroppenstedtii*-like species and the disease.

## Figures and Tables

**Table 1:** The primers employed in the amplification of *fusA* and *rpoB* genes.

Primers ID	Sequence	Position *
fusA401f	5′-GCTTCGTSAACAAGATGGAC-3′	401
fusA1516r	5′-RGTCTGCTTCTTGTGGGTG-3′	1516
C2700F	5′-CGWATGAACATYGGBCAGGT-3′	2714
C3130R	5′-TCCATYTCRCCRAARCGCTG-3′	3140

* The locus of primers is relative to that of the *Corynebacterium* spp. *fusA* and *rpoB* gene sequence [16,17].

**Table 2:** The demographic and medical data of the GLM patients.

Number	Age (year)	WBC (10^9/L)	CRP (mg/L)	The Size of the Main Abscess (cm)	Multiple Abscess	Enlargement of Axillary Lymph Nodes	Medical History
7403	30	no records	no records	7.0 × 6.0	No	Yes	Lactation
7413	39	12.91	no records	13.0 × 7.5	No	Yes	No obvious cause
7991	35	13.47	77.37	3.3 × 2.2	Yes	No	Two months after breast trauma
8117	39	13.8	43.15	3.8 × 1.0	No	No	No obvious cause
8226	31	14.81	20.96	0.7 (dilation of mammary ducts)	Yes	No	Being pregnant for 9 weeks
8285	37	11.51	4.33	10.7 × 3.6	Yes	Yes	No obvious cause
8318	34	6.14	6.58	5.4 × 1.5 × 3.4	No	No	No obvious cause

**Table 3:** ASTs results and minimum inhibitory concentration (MIC) values of the *C. kroppenstedtii*-like strains.

No of Isolates	Meropenem		Ciprofloxacin		Gentamicin		Clindamycin		Penicillin		Erythromycin		Vancomycin		Linezolid		Ceftriaxone	
	MIC	Result	MIC	Result	MIC	Result	MIC	Result	MIC	Result	MIC	Result	MIC	Result	MIC	Result	MIC	Result
7403	≤0.125	S	≥8	R	≤0.25	S	≥32	R	1.5	I	≥16	R	≤0.5	S	≤0.25	S	1	S
7413	≤0.125	S	0.064	S	≤0.25	S	≥32	R	0.125	S	≥16	R	≤0.5	S	≤0.25	S	0.5	S
7991	≤0.125	S	0.25	S	≤0.25	S	≥32	R	0.25	S	≥16	R	≤0.5	S	≤0.25	S	0.5	S
8117	0.5	I	≥8	R	≤0.25	S	≥32	R	0.125	S	≥16	R	≤0.5	S	≤0.25	S	2	I
8226	≤0.125	S	0.125	S	≤0.25	S	≥32	R	0.125	S	≥16	R	≤0.5	S	≤0.25	S	1	S
8285	≤0.125	S	0.125	S	≤0.25	S	≥32	R	0.5	I	≥16	R	≤0.5	S	≤0.25	S	0.5	S
8318	≤0.125	S	≥8	R	≤0.25	S	≥32	R	0.125	S	≥16	R	≤0.5	S	≤0.25	S	1	S

S: sensitive; I: intermediate; R: resistant.

**Table 4:** Vitek 2 ANC biochemical reaction test results of *C. kroppenstedtii-like* strains.

Substrate	7403	7413	7991	8117	8226	8285	8318
D-galactose	+	+	+	+	+	+	+
Leucine arylamidase	+	+	+	+	+	+	+
ELLMAN	+	+	+	+	+	+	+
Phenylalanine arylamidase	+	+	+	+	+	+	+
L-proline arylamidase	+	+	+	+	+	+	+
L-pyrrolydonyl-arylamidase	-	-	-	-	-	-	-
D-cellobiose	+	+	+	+	+	+	+
Tyrosine arylamidase	+	+	+	+	+	+	+
Ala-Phe-Pro arylamidase	+	-	-	-	-	-	-
D-glucose	+	+	+	+	+	+	+
D-mannose	+	+	+	+	+	+	+
D-maltose	+	+	+	+	+	+	+
Saccharose/sucrose	+	+	+	+	+	+	+
Arbutin	+	+	+	+	+	+	+
N-acetyl-D-glucosamine	+	+	+	+	+	+	+
5-bromo-4-chloro-3-indoxyl-β-glucoside	-	-	-	-	-	-	-
Urease	+	-	-	-	-	-	-
5-bromo-4-chloro-3-indoxyl-β-glucuronide	-	-	-	-	-	-	-
β-galactopyr anosidase indoxyl	-	-	-	-	-	-	-
α-arabinosidase	-	-	-	-	-	-	-
5-bromo-4-chloro-3-indoxyl-α-galactoside	-	-	-	-	-	-	-
β-mannosidase	-	-	-	-	-	-	-
Arginine	-	-	-	-	-	-	-
Pyruvate	-	-	-	-	-	-	-
Maltotriose	+	+	+	+	+	+	+
Esculin hydrolyse	-	-	-	-	-	-	-
β-D-fucosidase	-	-	-	-	-	-	-
5-bromo-4-chloro-3-indoxyl-β-N-acetyl-glucosamide	-	-	-	-	-	-	-
5-bromo-4-chloro-3-indoxyl-α-mannoside	-	-	-	-	-	-	-
α-L-fucosidase	-	-	-	-	-	-	-
Phosphatase	-	-	-	-	-	-	-
L-arabinose	+	-	+	+	+	+	-
D-ribose	+	-	+	+	+	+	-
Phenylphosphonate	-	-	-	-	-	-	-
α-L-arabinofur anosidase	-	-	-	-	-	-	-
D-xylose	+	-	+	+	+	+	-

+: The biochemical reaction is positive; -: the biochemical reaction is negative.

## Data Availability

The original contributions presented in the study are included in the article; further inquiries can be directed to the corresponding author.

## Supplementary Materials

The following supporting information can be downloaded at https://www.mdpi.com/article/10.3390/pathogens13100880/s1, Table S1. The most important *m*/*z* (Da) peaks of the new MSPs for *C. parakroppenstedtii.*

## References

[1] Kessler E., Wolloch Y. (1972). Granulomatous mastitis: A lesion clinically simulating carcinoma. Am. J. Clin. Pathol..

[2] Imoto S., Kitaya T., Kodama T., Hasebe T., Mukai K. (1997). Idiopathic granulomatous mastitis: Case report and review of the literature. Jpn. J. Clin. Oncol..

[3] Li X.Q., Yuan J.P., Fu A.S., Wu H.L., Liu R., Liu T.G., Sun S.R., Chen C. (2022). New Insights of *Corynebacterium kroppenstedtii* in Granulomatous Lobular Mastitis based on Nanopore Sequencing. J. Investig. Surg..

[4] Tariq H., Menon P.D., Fan H., Vadlamudi K.V., Pandeswara S.L., Nazarullah A.N., Mais D.D. (2022). Detection of *Corynebacterium kroppenstedtii* in Granulomatous Lobular Mastitis Using Real-Time Polymerase Chain Reaction and Sanger Sequencing on Formalin-Fixed, Paraffin-Embedded Tissues. Arch. Pathol. Lab. Med..

[5] Johnstone K.J., Robson J., Cherian S.G., Cheong J.W.S., Kerr K., Bligh J.F. (2017). Cystic neutrophilic granulomatous mastitis associated with Corynebacterium including *Corynebacterium kroppenstedtii*. Pathology.

[6] Wong S.C.Y., Poon R.W.S., Chen J.H.K., Tse H., Lo J.Y.C., Ng T.-K., Au J.C.K., Tse C.W.S., Cheung I.Y.Y., Yuk M.-T. (2017). *Corynebacterium kroppenstedtii* is an emerging cause of mastitis especially in patients with psychiatric illness on antipsychotic medication. Open Forum Infect. Dis..

[7] Bernard K. (2012). The genus corynebacterium and other medically relevant coryneform-like bacteria. J. Clin. Microbiol..

[8] Bernard K.A., Munro C., Wiebe D., Ongsansoy E. (2002). Characteristics of rare or recently described Corynebacterium species recovered from human clinical material in Canada. J. Clin. Microbiol..

[9] Collins M.D., Falsen E., Akervall E., Sjöden B., Alvarez A. (1998). *Corynebacterium kroppenstedtii* sp. nov., a novel corynebacterium that does not contain mycolic acids. Int. J. Syst. Bacteriol..

[10] Tauch A., Fernández-Natal I., Soriano F. (2016). A microbiological and clinical review on *Corynebacterium kroppenstedtii*. Int. J. Infect. Dis..

[11] Wong S.C.Y., Poon R.W., Foo C.H., Ngan A.H., Tse H., Lam V.C., Leung T.H., Wong C.P., Cheng V.C., Chen J.H. (2018). Novel selective medium for the isolation of *Corynebacterium kroppenstedtii* from heavily colonized clinical specimens. J. Clin. Pathol..

[12] Saraiya N., Corpuz M. (2019). *Corynebacterium kroppenstedtii*: A challenging culprit in breast abscesses and granulomatous mastitis. Curr. Opin. Obstet. Gynecol..

[13] Tan C., Lu F.I., Aftanas P., Tsang K.K., Mubareka S., Chan A., Kozak R. (2020). Whole genome sequence of *Corynebacterium kroppenstedtii* isolated from a case of recurrent granulomatous mastitis. IDCases.

[14] Luo Q., Chen Q., Feng J., Zhang T., Luo L., Chen C., Liu X., Xu N., Qu P. (2022). Classification of 27 *Corynebacterium kroppenstedtii*-Like Isolates Associated with Mastitis in China and Descriptions of *C. parakroppenstedtii* sp. nov. and *C. pseudokroppenstedtii* sp. nov. Microbiol. Spectr..

[15] Yu J., Zhou X.F., Yang S.J., Liu W.H., Hu X.F. (2013). Design and application of specific 16S rDNA-targeted primers for assessing endophytic diversity in Dendrobium officinale using nested PCR-DGGE. Appl Microbiol Biotechnol..

[16] Busse H.J., Kleinhagauer T., Glaeser S.P., Spergser J., Kämpfer P., Rückert C. (2019). Classification of three corynebacterial strains isolated from the Northern Bald Ibis (*Geronticus eremita*): Proposal of *Corynebacterium choanae* sp. nov., *Corynebacterium pseudopelargi* sp. nov., and *Corynebacterium gerontici* sp. nov. Int. J. Syst. Evol. Microbiol..

[17] Khamis A., Raoult D., La Scola B. (2004). rpoB gene sequencing for identification of Corynebacterium species. J. Clin. Microbiol..

[18] Li Y., Gu B., Liu G., Xia W., Fan K., Mei Y., Huang P., Pan S. (2014). MALDI-TOF MS versus VITEK 2 ANC card for identification of anaerobic bacteria. J. Thorac. Dis..

[19] Yoon S.H., Ha S.M., Lim J., Kwon S., Chun J. (2017). A large-scale evaluation of algorithms to calculate average nucleotide identity. Antonie Leeuwenhoek.

[20] Tamura K., Stecher G., Kumar S. (2021). MEGA11: Molecular Evolutionary Genetics Analysis Version 11. Mol. Biol. Evol..

[21] Binelli C., Lorimier G., Bertrand G., Parvery F., Bertrand A.F., Verriele V. (1996). Granulomatous mastitis and corynebacteria infection. Two case reports. J. Gynecol. Obstet. Biol. Reprod..

[22] Urbaniak C., Cummins J., Brackstone M., Macklaim J.M., Gloor G.B., Baban C.K., Scott L., O’Hanlon D.M., Burton J.P., Francis K.P. (2014). Microbiota of human breast tissue. Appl. Environ. Microbiol..

[23] Hieken T.J., Chen J., Hoskin T.L., Walther-Antonio M., Johnson S., Ramaker S., Xiao J., Radisky D.C., Knutson K.L., Kalari K.R. (2016). The microbiome of aseptically collected human breast tissue in benign and malignant disease. Sci. Rep..

[24] Taylor G.B., Paviour S.D., Musaad S., Jones W.O., Holland D.J. (2003). A clinicopathological review of 34 cases of inflammatory breast disease showing an association between corynebacteria infection and granulomatous mastitis. Pathology.

[25] Paviour S., Musaad S., Roberts S., Taylor G., Taylor S., Shore K., Lang S., Holland D. (2002). Corynebacterium species isolated from patients with mastitis. Clin. Infect. Dis..

[26] Boutet P., Sulon J., Closset R., Detilleux J., Beckers J.F., Bureau F., Lekeux P. (2007). Prolactin-induced activation of nuclear factor kappaB in bovine mammary epithelial cells: Role in chronic mastitis. J. Dairy Sci..

