# Supplementary material for: Clinical Identification of Two Novel C. kroppenstedtii-like Species Isolated as Pathogens of Granulomatous Lobular Mastitis
Source: Pathogens. 2024 Oct 9;13(10):880. doi: 10.3390/pathogens13100880 (PMC11514597; doi:10.3390/pathogens13100880)
Supplement: Supplementary file 1 [file pathogens-13-00880-s001.zip › pathogens-3170012-supplementary.pdf]

Table S1. The most important m/z(Da) peaks of the new MSPs for C.parakroppenstedtii.

m/z(Da)	Intensity(%)	Frequency(%) *
4206.86	1.73	83.3
6006.83	1.95	83.3
3158.25	2.92	88.9
3371.11	2.69	88.9
3699.22	2.31	88.9
4519.84	3.20	88.9
4619.33	11.74	88.9
6533.88	13.61	88.9
8413.42	1.71	88.9
8778.83	1.49	88.9
4061.68	3.11	94.4
4388.89	2.02	94.4
6168.22	3.49	94.4
7395.61	3.45	94.4
3291.42	47.94	100
4661.80	9.38	100
5607.93	6.46	100
5779.74	19.83	100
5826.54	6.19	100
6317.11	3.98	100
6582.79	100	100
6632.31	33.58	100

* All the frequencies of the peaks are higher than 80.0%.
